# Post Office and the Work of the Blind

**Published:** 1895-01-19

**Authors:** 


### THE POST OFFICE AND THE BLIND.

Some time ago, on the occasion of the distribution of prizes at the Institution for the General Welfare of the Blind, Mr. Burdett made some remarks in regard to the administration of the Post Office, pointing out that from what he had heard there was reason to believe that a system of blackmailing was in operation which made it impossible to obtain contracts for the supply of baskets to the Parcel Post Department except by the payment of gratuities to the subordinate officials.

On this the secretary to the Post Office, asked Mr. Burdett for particulars. To this letter Mr. Burdett replied, giving the name of his informant. He said, "In visiting the workshops, the Secretary, Captain Webber, R.N., stated that although they supplied some of the great railways with baskets, they did not tender for Post Office work, because to secure the work it was necessary to palm-oil the foreman or examiner who passed the baskets. He then gave me the grounds upon which this statement was made. I subsequently wrote to him for particulars, and I now beg to enclose you a copy of his letter containing them."

The letter of the Secretary contained the following: "The first lot of baskets sent in—quite a third—were rejected, but on Colonel Lewis going direct to the head of the department, and demanding to have the baskets inspected by him, the foreman admitted, on being brought to book, that he could find nothing against them, though he had previously returned them; showing pretty conclusively that, if Colonel Lewis had not fought against the injustice, he would have either to bribe the foreman to pass them, or have the baskets returned on his hands." To this the Post Office replied that this was not sufficient to support the allegation, adding, "Mr. Morley is confident that, unless you are able to produce some clear evidence of its truth, you will see fit to retract so serious a charge in as public a manner as you made it." Not unnaturally, Mr. Burdett declined to do anything of the sort. There was the statement made by responsible people, whose names were given; and, without pretending to investigate the matter, the Post Office asked that it should be withdrawn, apparently because it did not contain absolute proof of the charge. What Mr. Burdett wanted was investigation; he gave the hint, showed what was being said, and asked for inquiry. It was for the Post Office to prove or to disprove, not for Mr. Burdett, who, if he could have had proof in his pocket, would doubtless have framed his accusation in very different words. What he did was to ask for inquiry. The accusation had been made, Colonel Lewis was stated to have been to the Post Office and "fought against the injustice," and thus it seemed clear that the matter had been already brought under the official eye, and Mr. Burdett said, "What is demanded in the best interest of the public, the Post Office, and the Blind Institution is a full, impartial, and exhaustive inquiry into all the circumstances of the case."

When, ultimately, inquiry was made by Mr. Burdett, the accusation fell to the ground, and Mr. Burdett wrote, "I much regret that, on authority which I believed to be entirely reliable, I brought forward in my speech a complaint which my informant is now unable to stand by."

It is very satisfactory to find the Post Office cleared of a doubt which had been thrown upon the honour of its officers; it is not, however, so entirely satisfactory to observe the manner in which the complaint, obviously made in all honesty, was received by the department. The request that the complaint should be withdrawn without apparently any investigation, merely because it was not in the first instance proved up to the hilt, shows how ill public officials are apt to take hints which they ought to be most thankful to receive.

